# Time-Dependent Degradation of Imipenem in Aqueous Solution and Its Impact on Antibacterial Activity

**DOI:** 10.3390/antibiotics15070704

**Published:** 2026-07-20

**Authors:** Gu Hae Kim, Na Rae Kang, Jisoo Kang, Seungjae Jang, Seungyu Lee, Dawon Kang, Jeong Yoon Kim

**Affiliations:** 1 Department of Pharmaceutical Engineering, Institute of Agricultural and Life Science (IALS), Anti-Aging Bio Cell Factory Regional Leading Research Center (ABC-RLRC), Gyeongsang National University, Jinju 52828, Republic of Korea; kim9999hae@gnu.ac.kr (G.H.K.); rkdskfo0507@gnu.ac.kr (N.R.K.); ekfflrl8282@gnu.ac.kr (J.K.); 2026210163@gnu.ac.kr (S.J.); 220140128@gnu.ac.kr (S.L.); 2 Department of Physiology, College of Medicine, Gyeongsang National University, Jinju 52727, Republic of Korea; 3 Department of Convergence Medical Science and Institute of Medical Sciences, Gyeongsang National University, Jinju 52727, Republic of Korea

**Keywords:** Imipenem, stability, dimerization, LC-Q-TOF/MS analysis, antibacterial activity

## Abstract

**Background/Objectives**: Imipenem, known as a carbapenem, is widely used in combination with Cilastatin to treat severe bacterial infections. Imipenem is a β-lactam antibiotic with potent antibacterial activity but a relatively unstable chemical structure. Due to these structural characteristics, it may degrade or undergo structural changes over time in aqueous solutions. **Methods**: In this study, Imipenem was investigated for its structural stability over time in aqueous solution at 37 °C. Structural changes in the Imipenem/Cilastatin solution were confirmed by HPLC-DAD at the maximum absorbance wavelength. **Results**: Imipenem undergone progressive degradation and structural transformation for the dimerization, while Cilastatin remained stable under the same conditions. LC-Q-TOF/MS confirmed the formation of a dimeric Imipenem species after prolonged incubation. Antibacterial activity showed that Imipenem was effective against *P. carotovorum* regardless of the dissolution times. However, Imipenem solution after 24 h with chemical degradation exhibited significantly decreased effects against *E. coli* and *P. aeruginosa*. **Conclusions**: Accordingly, the patterns of metabolite changes in Imipenem and Cilastatin were characterized according to concentration and dissolution time, and the corresponding antibacterial activities against different bacterial strains were compared.

## 1. Introduction

Imipenem is one of the most widely used carbapenem antibiotics for the treatment of severe bacterial infections [1,2]. However, its chemical instability in aqueous solution has long been recognized as a limitation affecting drug preparation and therapeutic efficacy [3,4]. Imipenem is a broad-spectrum beta-lactam antibiotic belonging to the carbapenem class. It is highly effective against severe bacterial infections caused by a wide range of organisms, including Gram-positive, Gram-negative, aerobic, and anaerobic bacteria [5,6]. Carbapenems, including Imipenem, are particularly valuable because they are resistant to most beta-lactamase enzymes produced by multidrug-resistant bacteria, making them a key option for treating difficult infections [7,8,9]. However, Imipenem alone is rapidly inactive in the kidneys by an enzyme called renal dehydropeptidase I (DHP-I) [10,11]. This enzymatic degradation limits its effectiveness and shortens its duration of action [12].

Cilastatin, a renal DHP-I inhibitor, plays a key role in enhancing the efficacy and safety of Imipenem [13]. This allows Imipenem to remain active in the body for extended periods, maximizing its antibacterial effect. Cilastatin protects Imipenem from degradation and reduces the risk of nephrotoxicity [14,15]. It is used to inhibit the renal organic anion transporter (OAT), which is involved in the absorption of nephrotoxic substances in the kidney [16,17]. Co-administration of Cilastatin and Imipenem allows Imipenem to act in the bloodstream and tissues, making it particularly effective against severe bacterial infections caused by infectious microorganisms [5,18,19].

Imipenem/Cilastatin is a critical medication used to treat a wide range of serious bacterial infections, including pneumonia, sepsis, endocarditis, intra-abdominal infections, osteomyelitis arthritis, septic arthritis, and complicated urinary tract infections [20,21,22]. Imipenem and Cilastatin combined are used to treat complicated infections caused by multidrug-resistant bacteria in various parts of the body [13]. This combination is particularly effective because it addresses both the antibacterial action and the stability of the drug in the body [23].

The β-lactam moiety in Imipenem is the primary active component of the structure, but it is also a highly interrelated component. The β-lactam skeleton in the elbow is hydrolytic and readily degrades over time. Therefore, Imipenem undergoes rapid degradation in storage, such as in an injectable form [24,25,26]. Although the aqueous instability of Imipenem has been well documented, previous studies have primarily focused on degradation kinetics, pharmaceutical stability during storage, or analytical identification of degradation products [3,4,27]. While these studies have substantially improved our understanding of Imipenem degradation, it remains unclear whether time-dependent structural transformations directly influence antibacterial activity and whether these effects differ among bacterial species. Furthermore, only a limited number of studies have attempted to integrate physicochemical characterization with microbiological evaluation to establish a structure–function relationship during Imipenem degradation.

Therefore, the present study aimed to comprehensively investigate the time-dependent structural transformation of Imipenem in aqueous solution using complementary analytical techniques, including HPLC-DAD, UV spectroscopy, and LC-Q-TOF/MS, and to evaluate whether these structural changes influence antibacterial activity against representative bacterial strains. By correlating molecular transformation with biological activity, this study provides practical information for the appropriate preparation and handling of aqueous Imipenem solutions.

## 2. Results and Discussion

### 2.1. Visual Changes of Imipenem/Cilastatin Solutions

**Figure 1:**
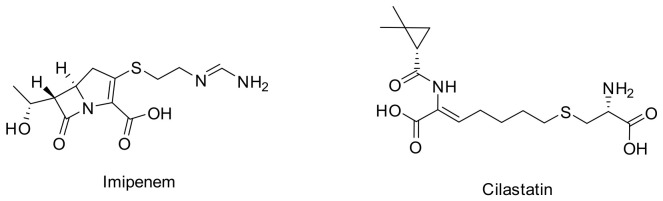
Chemical structure of Imipenem and Cilastatin.

**Figure 2:**
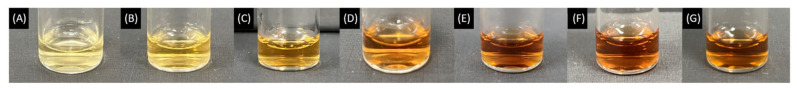
Observation of Imipenem/Cilastatin solution (5000 μg/mL) at different dissolution intervals at (**A**) 0.5 h, (**B**) 1 h, (**C**) 3 h, (**D**) 6 h, (**E**) 18 h, (**F**) 24 h, and (**G**) 48 h.

Imipenem is a carbapenem β-lactam antibiotic possessing a fused bicyclic β-lactam/carbapenem core. Cilastatin is a renal dehydropeptidase-I (DHP-I) inhibitor that prevents the enzymatic hydrolysis of Imipenem in the kidney, thereby prolonging its antibacterial activity (Figure 1). Imipenem is usually used together with Cilastatin to be prescribed in an average ratio of 1:1 with the concentrations at 5~10 mg/mL [12]. Thus, Imipenem/Cilastatin solutions were prepared at a concentration of 5 mg/mL, corresponding to a clinically relevant preparation concentration. Figure 2 presented Imipenem/Cilastatin solutions (5 mg/mL), which showed a progressive color change from initially light yellow to gradually darkening and eventually brown with increasing dissolving times.

The progressive discoloration observed in the aqueous Imipenem solution may reflect an underlying chemical transformation occurring during prolonged incubation. Although visual observation alone cannot confirm degradation, the observed color change was consistent with analytical results from HPLC-DAD, UV spectroscopy, and LC-Q-TOF/MS, all of which demonstrated time-dependent structural changes in Imipenem. Similar discoloration associated with β-lactam degradation has been reported previously [27,28].

### 2.2. HPLC Analysis of Imipenem/Cilastatin Solutions

**Figure 3:**
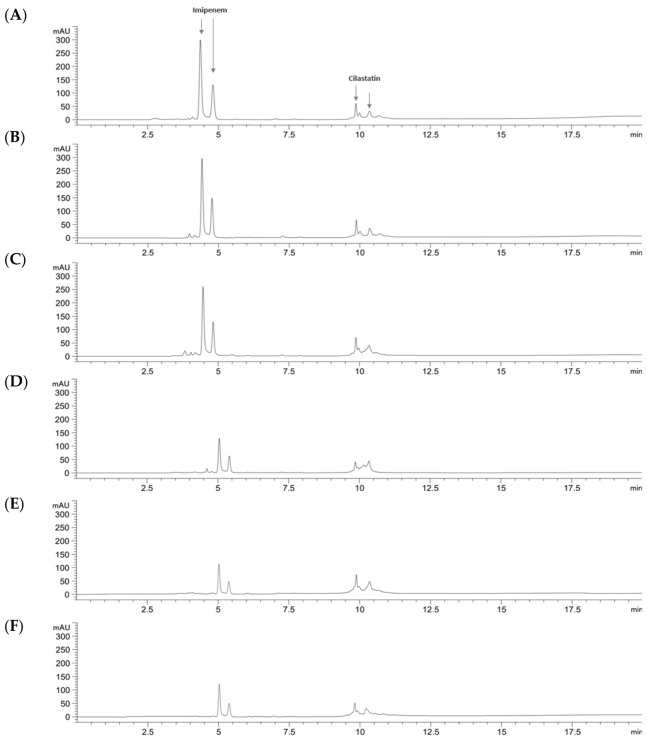
HPLC patterns (297 nm) of Imipenem/Cilastatin solution by various dissolution intervals at (**A**) 0.5 h, (**B**) 1 h, (**C**) 6 h, (**D**) 18 h, (**E**) 24 h, and (**F**) 48 h.

**Figure 4:**
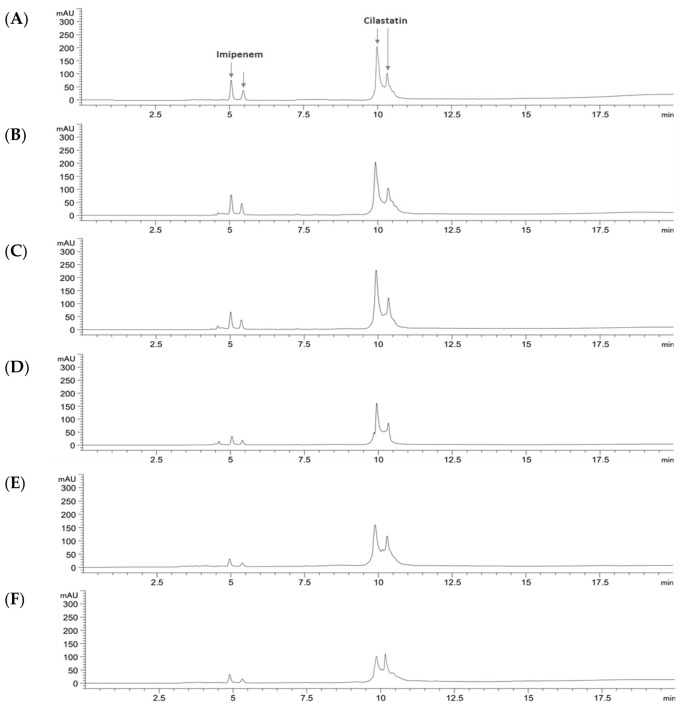
HPLC patterns (243 nm) of Imipenem/Cilastatin solution by various dissolution intervals at (**A**) 0.5 h, (**B**) 1 h, (**C**) 6 h, (**D**) 18 h, (**E**) 24 h, and (**F**) 48 h.

To further evaluate the time-dependent stability of Imipenem and Cilastatin, the aqueous solution was analyzed by HPLC-DAD at 297 nm and 243 nm, corresponding to the maximum absorbance wavelengths of Imipenem and Cilastatin, respectively. Since a diode-array detector was employed, chromatograms at both wavelengths were simultaneously obtained from the same sample injection. As shown in Figure 3 and Figure 4, the chromatographic profile of Cilastatin remained essentially unchanged throughout the incubation period, indicating that Cilastatin was chemically stable under the experimental conditions. Although a weak Cilastatin signal was also detected in the chromatograms monitored at 297 nm, no significant change in its retention time or peak profile was observed at the optimal detection wavelength (243 nm). Likewise, degradation-related peaks of Imipenem were predominantly detected at 297 nm because they retained the characteristic absorbance of Imipenem, whereas they exhibited little or no absorbance at 243 nm. In contrast, the Imipenem peak gradually decreased with increasing dissolution time, accompanied by changes in both peak intensity and retention behavior. During the early incubation period (0.5–6 h), the major Imipenem peak eluted at approximately 4.3–4.8 min. However, after prolonged incubation (18–48 h), additional peaks appeared at slightly longer retention times, accompanied by a concomitant decrease in the original Imipenem peak. These chromatographic changes suggest that Imipenem underwent progressive structural transformation during incubation in aqueous solution. These chromatographic findings were further supported by UV spectroscopic and LC-Q-TOF/MS analyses, collectively indicating time-dependent structural transformation of Imipenem in aqueous solution.

### 2.3. UV Spectroscopic Analysis of Imipenem

**Figure 5:**
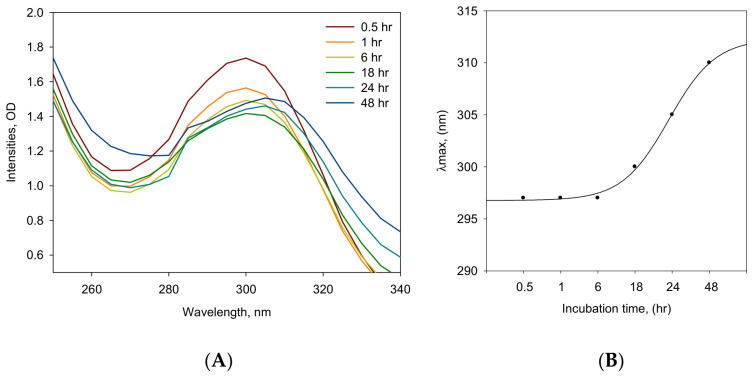
(**A**) UV spectra of Imipenem and (**B**) maximal absorbance wavelength by different solution time intervals.

Previous results suggested possible chemical transformation of Imipenem, including the visual evidence and HPLC chromatograms in the solutions. Therefore, UV spectroscopic analysis was performed to observe changes in the chemical properties of Imipenem. In Figure 5A, the UV scan of an analytical standard Imipenem confirmed that the maximal absorption wavelength changed as the dissolution time changed. From 0.5 to 6 h, the maximum absorption wavelength remained constant at 297 nm, then it gradually shifted to longer wavelengths (Figure 5B). Specifically, 18 h, 24 h, and 48 h after dissolution had λmax of 297, 305, and 310 nm. These results indicate that spectral changes began after approximately 18 h, with a distinct bathochromic shift becoming evident at 24 and 48 h. After the points, Imipenem had a distinct chromophore compared to before reflecting changes in the electronic structure associated with molecular transformation.

### 2.4. LC-Q-TOF/MS Analysis of Imipenem

**Figure 6:**
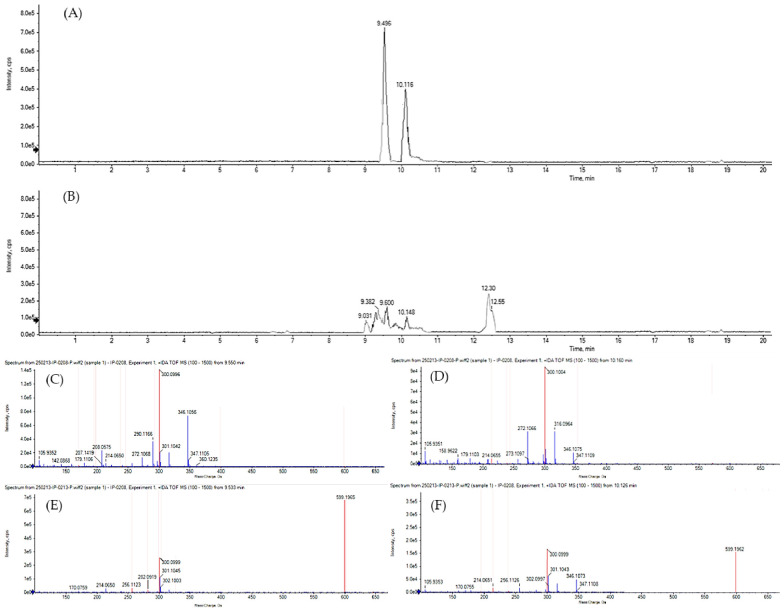
BPC (base peak chromatogram) of Imipenem in aqueous solution at different incubating times with (**A**) 0.5 h and (**B**) 48 h by LC-Q-TOF/MS analysis. (**C**–**F**) Individual mass grams of representative peaks in 0.5 h incubating Imipenem (*t*_R_ = 9.496, (**C**); *t*_R_ = 10.116, (**D**)) and 48 h incubating Imipenem (*t*_R_ = 12.30, (**E**); *t*_R_ = 12.38, (**F**)).

**Figure 7:**
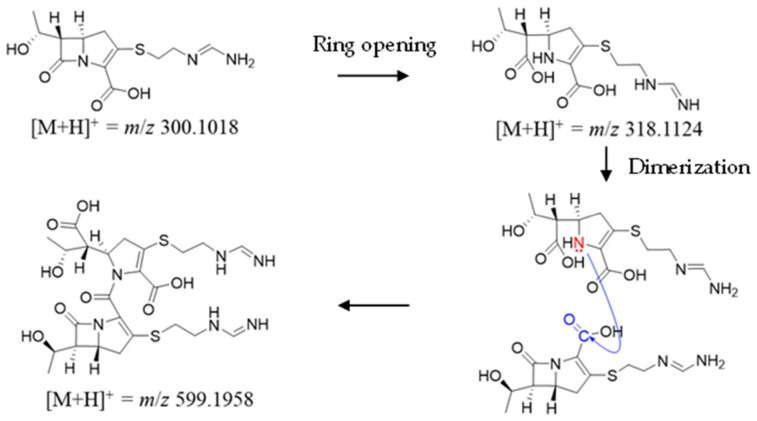
Expected chemical structural changes procedure of Imipenem over time according to observed mass from LC-Q-TOF/MS analysis.

LC-Q-TOF/MS analysis was conducted in positive ion mode to identify the structural changes in Imipenem over time, specifically. Figure 6A,B displayed the base peak chromatogram (BPC) of Imipenem solution immediately after dissolution and 48 h after dissolution, respectively. Immediately after dissolution, two closely eluting peaks were observed at retention times of 9.5 and 10.1 min (Figure 6A). Both peaks exhibited nearly identical protonated molecular ions ([M+H]^+^) at *m*/*z* 300.0996 and 300.1004, respectively (Figure 6C,D), which were in good agreement with the theoretical mass of Imipenem (C_12_H_17_N_3_O_4_S). MS/MS spectra were acquired for both precursor ions (Figure S1). The two precursor ions exhibited highly similar fragmentation patterns, with common product ions observed at *m*/*z* 98.006, 126.038, 141.995, 170.027, and 214.066. Together with their nearly identical accurate masses, these findings support the interpretation that the two peaks represent closely related molecular species rather than time-dependent degradation products, because both peaks were already detected immediately after dissolution. However, the available MS/MS data were insufficient to unambiguously determine their exact structural relationship. After 48 h of dissolution, chromatographed peak patterns changed significantly. Multiple peaks appeared between the retention times of 9~10 min, and two dominant peaks (*t*_R_ = 12.3 and 12.6 min) were shown (Figure 6B). The observed mass at the main peaks (*t*_R_ = 12.3 and 12.6 min) showed at [M+H]^+^ = *m*/*z* 599.1965 and 599.1962, respectively. The observed ion at *m*/*z* 599 was consistent with the expected [2M−H_2_O+H]^+^ ion formed by condensation of two Imipenem molecules. The predicted chemical formula for a dimeric form of Imipenem corresponded with the theoretical mass of C_24_H_34_N_6_O_8_S_2_, having an error of +1.16 and +0.67 ppm compared to the detected ion mass. The mass error for both peaks was within ±2 ppm, supporting the reliability of the proposed elemental composition. Based on the results, the predicted pathway suggests that the dimerization occurs via the reaction between the amine group (-NH_2_) of the β-lactam ring and the carboxylic acid group (-COOH), resulting in amide formation as compared with the literature [29]. Accordingly, a putative dimerization pathway is proposed in Figure 7. The ring opening of Imipenem may generate a free amino group that subsequently reacts with the carboxylic acid group of another Imipenem molecule to form a dimeric species. Collectively, these findings suggest that Imipenem undergoes a time-dependent structural transformation in aqueous solution, leading to the formation of a putative dimeric species via self-condensation. Previous studies have reported that Imipenem undergoes hydrolysis and oligomerization in aqueous solution, yielding several degradation products that depend on storage conditions and formulation [4,28]. LC-Q-TOF/MS analysis was generally consistent with these observations, demonstrating the formation of a dimeric species after prolonged incubation. However, rather than focusing solely on structural characterization of degradation products, the present study further evaluated the biological significance of these molecular changes by correlating structural transformation with antibacterial activity.

### 2.5. Antibacterial Effects of Imipenem by Time Intervals

**Figure 8:**
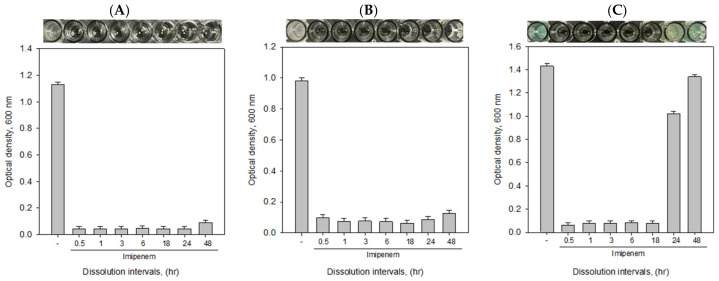
Changes on antibacterial effects of Imipenem by different dissolution intervals (0.5, 1, 3, 6, 18, 24, and 48 h) against (**A**) *Pectobacterium carotovorum*, (**B**) *Escherichia coli*, and (**C**) *Pseudomonas aeruginosa.*

To further investigate the effects of Imipenem on antibacterial efficacy, Imipenem was incubated for 0, 1, 6, 18, 24, and 48 h, and the resulting solutions were tested against three representative bacterial strains, including *P. carotovorum*, *E. coli*, and *P. aeruginosa* (Figure 8). For *P. carotovorum*, the Imipenem solution retained apparent antibacterial activity across all tested dissolution times from freshly prepared up to 48 h. It confirmed that both the normal and dimeric forms of Imipenem had practical antibacterial activity against *P. carotovorum*. In contrast, the antibacterial activity of Imipenem against *E. coli* showed a slightly time-dependent decrease. While solutions incubated for up to 24 h retained substantial inhibitory effects, antibacterial potential was slightly reduced in the 48 h solution. The most pronounced time-dependent loss of antibacterial effects was observed with *P. aeruginosa*. Freshly prepared Imipenem solutions or those incubated for up to 18 h demonstrated significant antibacterial activity. However, solutions incubated for 24 h or longer showed a marked decrease in effectiveness, with the 48 h solution exhibiting almost a complete loss of activity against this strain. The diminished efficacy is likely attributable to the time-dependent structural transformation of Imipenem, resulting in a lower abundance of the active form available to inhibit bacterial growth. These results demonstrated that the stability of Imipenem in aqueous solutions also affects its antibacterial activity, with inhibition rates varying across bacterial strains. Additionally, the time between solution preparation and administration is important when using Imipenem against pathogens with low susceptibility. Unlike previous studies that primarily investigated degradation kinetics or analytical characterization of degradation products, the present work establishes a direct association between molecular transformation and antibacterial efficacy. This integrated analytical–microbiological approach provides additional insight into the practical implications of Imipenem degradation and may serve as a useful framework for evaluating the functional consequences of instability in other β-lactam antibiotics.

## 3. Materials and Methods

### 3.1. Preparation of Imipenem/Cilastatin Solutions

An experimental combined chemical of Imipenem and Cilastatin was purchased from Asta Tech, Inc. (Bristol, PA, USA). An Imipenem/Cilastatin stock solution (5 mg/mL) was prepared in distilled water. The stock solution was subsequently diluted with distilled water, as required for each experiment. The temperature of Imipenem/Cilastatin solution was kept at 37 °C using a Heating block/Dry bath (MaxtableTM H10, DAIHAN Scientific, Wonju-si, Gangwon-do, Republic of Korea). The prepared Imipenem/Cilastatin solutions (5000 µg/mL) were observed by various dissolution time intervals at 0.5, 1, 3, 6, 18, 24, and 48 h.

### 3.2. HPLC Analysis of Imipenem/Cilastatin Solutions

A concentration at 5000 µg/mL of Imipenem/Cilastatin solutions was obtained with different dissolution times of 0.5, 1, 3, 6, 18, 24, and 48 h, maintaining a temperature at 37 °C using a heating block. The stock solution was diluted with distilled water to obtain a final concentration of 1000 µg/mL for HPLC analysis. The diluted samples were filtered using 0.2 µm syringe filter to put in HPLC vial. The stability and solubility of the Imipenem/Cilastatin solution over time were evaluated using an Agilent 1200 series system with a binary pump, auto sampler, column oven, and diode array detector (Agilent Technologies, Santa Clara, CA, USA). The filtered solutions (10 µL) were injected on the column (Supersil 120 ODS-I column, 4.6 × 250 mm, 5 µm, Agilent Technologies, Santa Clara, CA, USA). The elution solvent was used as a combination of mobile phase A (water containing 0.1% acetic acid) and B (acetonitrile containing 0.1% acetic acid). A gradient LC pump condition at a flow rate of 1 mL/min was as follows: 0 min, 5% B; 5 min, 10% B; 8 min, 75% B; 15 min, 100% B. The observed wavelengths were 243 nm (the maximal wavelength of Cilastatin) and 297 nm (the maximal wavelength of Imipenem). LC chromatogram was obtained through Chemstation software (version B.04.03, Agilent Technologies, Santa Clara, CA, USA).

### 3.3. Ultraviolet (UV) Analysis of Imipenem Solutions

A representative sample of Imipenem solution at 5000 µg/mL was collected for the UV analysis with the dissolution times of 0.5, 1, 3, 6, 18, 24, and 48 h at 37 °C. Different times with Imipenem/Cilastatin solution (5000 µg/mL) was 30 times diluted as 200 µg/mL to fill in a Quartz Cell Cuvette. UV analysis was conducted using spectrophotometer (iD3 SpectraMax, Molecular Devices, San Jose, CA, USA). The UV data acquisition was processed using SoftMax Pro 5.4.1 software with spectrum read modes. The UV spectrum was set to a wavelength range from 230 nm to 400 nm, which allows the detection of Imipenem.

### 3.4. LC-Q-TOF/MS Analysis of Imipenem Solutions

The analytical standard Imipenem was injected into LC-Q-TOF/MS, equipped with LC (Shimadzu NEXERA, Kyoto, Japan) and Q-TOF/MS (SCIEX X500R Q-TOF, Framingham, MA, USA). Imipenem (Sigma Aldrich, St. Louis, MO, USA) was prepared at concentrations of 1000 µg/mL and incubated for 30 min to 48 h. Both Imipenem solutions were filtered using a 0.2 µm syringe filter to pass over an InfinityLab Poroshell 120 HILIC column (4.6 × 150 mm, 4 µm, Agilent Technologies, Santa Clara, CA, USA). The solvent system was applied to a gradual condition with the mobile phase A (water with 0.1% acetic acid) and B (acetonitrile with 0.1% acetic acid) as follows: 0 min, 10% B; 5 min, 30% B; 10 min, 35% B; 20 min, 100% B. The flow rate was set at 0.5 mL per minute. The mass spectrometry was used in positive ionization mode with a temperature of 450 °C in electrospray ionization (ESI) mode. The capillary voltage and collision energy were 5.5 kV and 10 V, respectively. The desolvation gas flow was kept at 800 L/h. The mass value with mass-to-charge (*m*/*z*) ratio displayed the scan range from 50 to 1000. MS/MS spectra were acquired using information-dependent acquisition (IDA) mode with a collision energy of 35 V.

### 3.5. Antibacterial Effects of Imipenem Solutions

*Pseudomonas aeruginosa*, *Escherichia coli*, and *Pectobacterium carotovorum* were obtained from the Korean Agricultural Culture Collection (KACC, Wanju, Republic of Korea). All bacterial strains were cultured in Luria–Bertani (LB) broth (Difco™ LB Broth, Miller, BD, Franklin Lakes, NJ, USA) at 30 °C for 48 h. The bacterial suspensions were adjusted to approximately 1 × 10^8^ CFU/mL before the antibacterial assay. Imipenem/Cilastatin solution (1000 µg/mL) was incubated in distilled water for 0.5, 1, 3, 6, 18, 24, and 48 h. For each assay, 10 µL of bacterial suspension was added to 180 µL of LB broth and mixed with 10 µL of the Imipenem/Cilastatin solution in a 96-well microplate, followed by incubation at 30 °C for 48 h. Bacterial growth in wells without Imipenem/Cilastatin served as the untreated control.

## 4. Conclusions

In this study, we demonstrated that Imipenem undergoes progressive time-dependent structural transformation in aqueous solution at 37 °C. The structural changes in Imipenem were confirmed at 37 °C. Visual observation of the aqueous solution showed that its color gradually changed with time. Furthermore, the HPLC chromatogram at the maximal absorption wavelength (297 nm) of Imipenem confirmed that the peak retention time began to change approximately 18 h after dissolution. In contrast, Cilastatin showed almost no change in retention time or content over time at 243 nm (the maximum absorption wavelength), exhibiting relatively stable characteristics. The UV absorption spectrum remained essentially unchanged up to 18 h. However, spectral changes became apparent after this time, and a bathochromic shift of the maximum absorbance wavelength was clearly observed at 24 and 48 h. Furthermore, LC-Q-TOF/MS analysis revealed a distinct change in peak patterns between the aqueous solution immediately after dissolution and after 48 h, and the observed mass values confirmed the possibility of the formation of the dimeric form of Imipenem. Based on these structural changes in Imipenem, antimicrobial activity against *P. carotovorum*, *E. coli*, and *P. aeruginosa* was evaluated. The results showed that antimicrobial activity tended to be maintained regardless of dissolution time against *P. carotovorum* and *E. coli*. However, against *P. aeruginosa*, antimicrobial activity decreased significantly from 18 h onward, when the dimeric form began to form. Therefore, it is concluded that structural changes in the aqueous Imipenem solution at 37 °C may affect antimicrobial activity, and appropriate management of preparation and use time is necessary to maintain optimal antimicrobial efficacy. From a practical perspective, these findings suggest that prolonged storage of aqueous Imipenem solutions may not only reduce drug concentration but also alter antibacterial performance, depending on the target pathogen. Therefore, evaluation of antibiotic stability should include not only physicochemical characterization but also biological assessment to better predict therapeutic performance after preparation.

## Data Availability

The data presented in this study are available from the corresponding author upon reasonable request due to privacy and ethical restrictions.

## Supplementary Materials

The following supporting information can be downloaded at: https://www.mdpi.com/article/10.3390/antibiotics15070704/s1, Figure S1. MS/MS spectra of the two closely eluting precursor ions observed in the base peak chromatogram (BPC) are shown in Figure 6A. The spectra correspond to precursor ions detected at (A) RT = 9.55 min and (B) RT = 10.16 min.
