# Supplementary material for: Time-Dependent Degradation of Imipenem in Aqueous Solution and Its Impact on Antibacterial Activity
Source: Antibiotics (Basel). 2026 Jul 20;15(7):704. doi: 10.3390/antibiotics15070704 (PMC13406044; doi:10.3390/antibiotics15070704)
Supplement: Supplementary file 1 [file antibiotics-15-00704-s001.zip › antibiotics-4349195-supplementary.pdf]

(A)

(B)

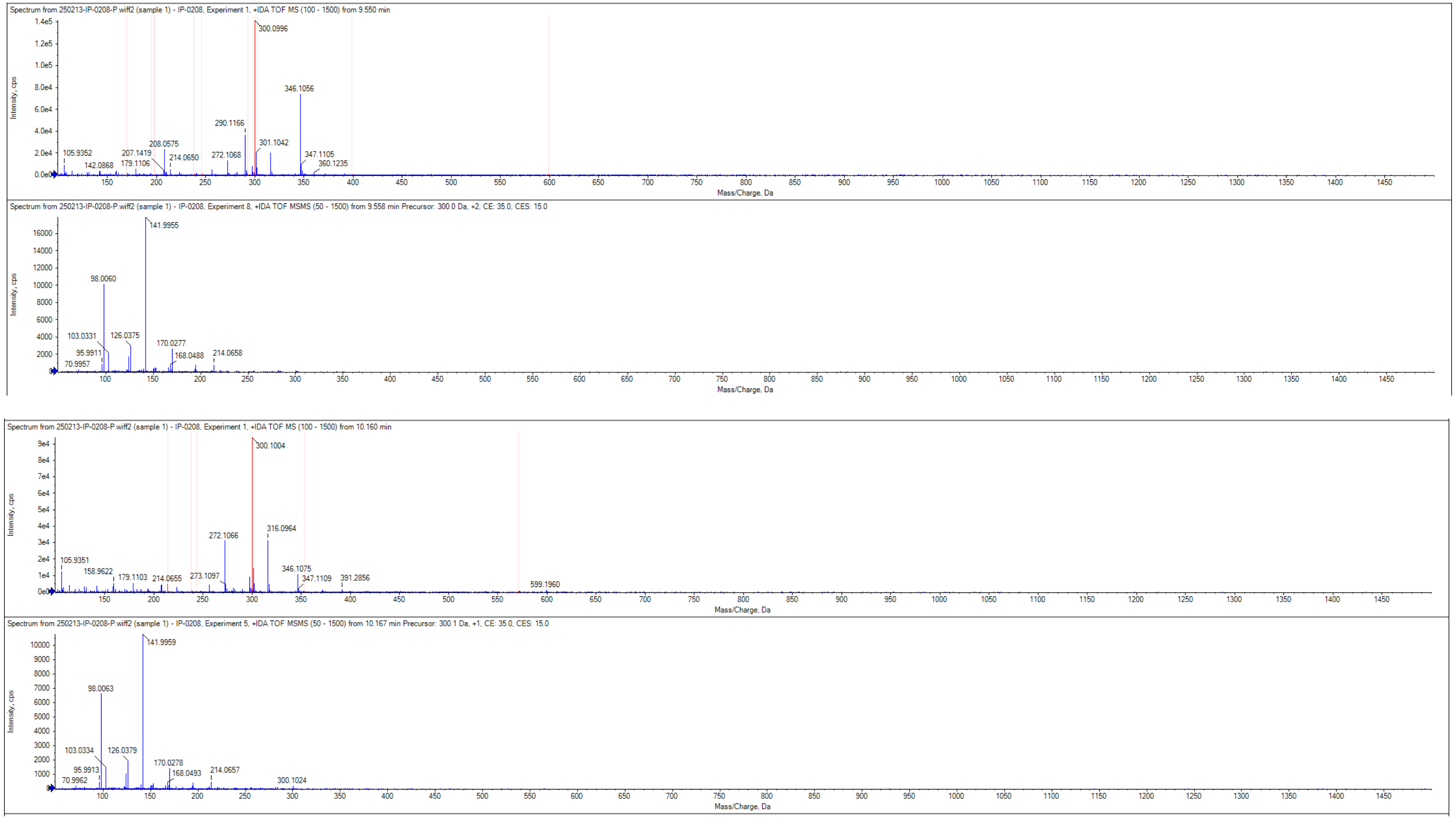

Figure S1. MS/MS spectra of the two closely eluting precursor ions observed in the base peak chromatogram (BPC) are shown in Figure 6A. The spectra correspond to precursor ions detected at (A) RT = 9.55 min and (B) RT = 10.16 min.
